# Biochemical markers of bone turnover in patients with spinal metastases after resistance training under radiotherapy – a randomized trial

**DOI:** 10.1186/s12885-016-2278-1

**Published:** 2016-03-17

**Authors:** Harald Rief, Georg Omlor, Michael Akbar, Thomas Bruckner, Stefan Rieken, Robert Förster, Ingmar Schlampp, Thomas Welzel, Tilman Bostel, Heinz Jürgen Roth, Jürgen Debus

**Affiliations:** Department of Radiation Oncology, University Hospital of Heidelberg, Im Neuenheimer Feld 400, 69120 Heidelberg, Germany; Department of Medical Biometry, University Hospital of Heidelberg, Im Neuenheimer Feld 305, 69120 Heidelberg, Germany; Department of Orthopaedics and Trauma Surgery, University Hospital of Heidelberg, Schlierbacherstrasse 120a, 69118 Heidelberg, Germany; Department of Endocrinology/Oncology, Limbach Laboratory Heidelberg, Im Breitspiel 15, 69126 Heidelberg, Germany

**Keywords:** Bone metastases, Spine, Physical exercise, Biochemical markers, Resistance training

## Abstract

**Background:**

To compare the effects of resistance training versus passive physical therapy on bone turnover markers (BTM) in the metastatic bone during radiation therapy (RT) in patients with spinal bone metastases. Secondly, to evaluate an association of BTM to local response, skeletal-related events (SRE), and number of metastases.

**Methods:**

In this randomized trial, 60 patients were allocated from September 2011 to March 2013 into one of the two arms: resistance training (Arm A) or passive physical therapy (Arm B) with thirty patients in each arm during RT. Biochemical markers such as pyridinoline (PYD), desoxy-pyridinoline (DPD), bone alkaline phosphatase (BAP), total amino-terminal propeptide of type I collagen (PINP), beta-isomer of carboxy-terminal telopeptide of type I collagen (CTX-I), and cross-linked N-telopeptide of type I collagen (NTX) were analyzed at baseline, and three months after RT.

**Results:**

Mean change values of PYD and CTX-I were significantly lower at 3 months after RT (*p* = 0.035 and *p* = 0.043) in Arm A. Importantly, all markers decreased in both arms, except of PYD and CTX-I in arm B, although significance was not reached for some biomarkers. In arm A, the local response was significantly higher (*p* = 0.003) and PINP could be identified as a predictor for survivors (OR 0.968, 95%CI 0.938–0.999, *p* = 0.043). BAP (OR 0.974, 95%CI 0.950–0.998, *p* = 0.034) and PINP (OR 1.025, 95%CI 1.001–1.049, *p* = 0.044) were related with an avoidance of SRE.

**Conclusions:**

In this group of patients with spinal bone metastases, we were able to show that patients with guided resistance training of the paravertebral muscles can influence BTM. PYD and CTX-I decreased significantly in arm A. PINP can be considered as a complementary tool for prediction of local response, and PINP as well as BAP for avoidance of SRE.

**Trial registration:**

Clinical trial identifier NCT 01409720. August 2, 2011.

## Background

Spinal bone metastases represent the most frequent site of skeletal metastasis [1], and radiotherapy (RT) is the most common treatment option of bone metastases in an advanced tumour disease [2]. As a result of the alteration in bone remodeling activity in patients with bone metastases, various markers of bone formation or resorption have been investigated as a measurement of activity of skeletal metastases. Balance between bone resorption and bone formation is required for maintenance of bone metabolism, and can be reflected by bone turnover markers (BTM). The usefulness of BTM as a tool for the diagnosis of bone metastases in many types of cancers has been investigated previously [3]. Previous studies have demonstrated that some BTM can indicate the existence of bone metastases [4], and have analyzed their relationship with clinical outcomes [5–9].

The two most clinically relevant markers of bone turnover in patients with skeletal metastases are bone-specific alkaline phosphatase (BAP) and N-terminal telopeptide of collagen type I (NTX). Total amino-terminal propeptide of type I collagen (PINP) and BAP are markers of bone formation; BAP has a linear relationship with osteoblast and osteoblastic precursor activity. NTX is a breakdown product of type I collagen produced by various proteolytic enzymes during bone resorption and dissolution of the organic bone matrix. Additionally, pyridinium cross-links pyridinoline (PYD) and desoxy-pyridinoline (DPD) are well-characterized markers for bone resorption. C-terminal cross-linking telopeptide of type I collagen (CTX-I) can be considered a complementary tool for prediction of clinical outcome as a marker of resorption. At the present time, although biochemical markers of bone turnover have shown some utility in clinical trials by predicting clinical outcomes such as death or development of SREs, they are not routinely used in clinical practice. Questions remain as to how clinicians should best use these markers to select and time appropriate treatments among patients with bone metastases. The use of these markers may, in the future, better allow physicians to selectively treat patients with bone metastases.

In our recent work, we could show the feasibility of resistance training in patients with spinal bone metastases under RT [10]. Importantly, we demonstrated that the bone density in metastases of the resistance training arm was significantly higher than in the control arm after 3 months. Therefore, we hypothesized that resistance training concomitant to RT could influence bone metabolism, and would be reflected by BTM.

The aim of this randomized trial was to compare the effect of resistance training concomitant to RT versus RT only on BTM, such as BAP, PINP, CTX-I, PYD, DPD, and NTX in patients with spinal bone metastases. Secondly, we evaluated an association of these markers as predicted factors for local response, prevention of SRE, and number of metastases.

## Methods

### Settings and patients

This is a randomized, controlled, two-armed intervention trial. A block randomization approach with block size 6 was used to ensure that the two intervention groups were balanced. After the baseline measurements, the patients with stable bone metastases were assigned to the respective treatment groups on a 1:1 basis according to the randomization list. Arm A (intervention group, resistance training) and arm B (control group, passive physical therapy) each consisted of 30 patients.

The blood and urine parameters were measured before start of RT (t_0_) (day of the first fraction), and after three months (t_2_) on an empty stomach. The primary endpoint was to compare BTM after 3 months in patients treated with resistance training concomitant to RT versus RT only in spinal bone metastases. Secondary endpoint was to evaluate predictive bone markers for local response, prevention of SRE, and number of metastases. Local response of metastasis was assessed on the basis of computed tomography. Positive local response was defined as complete or partial re-calcification on the basis of computed tomography observation at 3 months after RT. The definition of SRE was the first of any of the following events: pathological fractures, severe pain (increase of more than 2 points according to numeric rating scale), hypercalcaemia, and spinal cord compression. Number of metastases was classified in 1 or >1 (solitary vs. multiple). The data of the patient records were collected by the authors. Patient characteristics are shown in Table 1.

**Table 1:** Patient characteristics at baseline

		Intervention group (*n* = 30)		Control group (*n* = 30)	
		*n*	%	*n*	%
Age (years)					
	Mean (SD)	61.3 +/− 10.1		64.1 +/− 10.9	
Gender					
	Male	14	46.7	19	63.3
	Female	16	53.3	11	36.7
Karnofsky-index (median, range)		80 (70–100)		80 (70–100)	
Primary site					
	Lung cancer	12	9.2	8	26.6
	Breast cancer	5	16.7	6	20.1
	Prostate cancer	5	16.7	9	30.1
	Melanoma	1	3.3	1	3.3
	Renal cancer	1	3.3	2	6.7
	Other	6	20.1	4	13.4
Localization metastases					
	Thoracic	17	56.7	14	46.7
	Lumbar	9	30.0	13	43.3
	Thoracic and lumbar	2	6.7	2	6.7
	Sacrum	2	6.7	1	3.3
Number metastases					
	Mean (range)	1.4 (2–4)		1.7 (1–5)	
	Solitary	22	73.3	18	60.0
	Multiple	8	26.7	12	40.0
Type of metastases					
	osteoblast	9	30.0	10	33.3
	osteolytic	21	70.0	20	66.7
Size of metastasis					
	Mean (SD)	318.6 +/− 230.0		380.7 +/− 193.6	
Distant metastases at baseline					
	Visceral	12	40.0	5	16.7
	brain	3	10.0	3	10.0
	lung	7	23.3	4	13.3
	tissue	8	26.7	6	20.0
Hormonotherapy		10	33.3	16	53.3
Immunotherapy		7	23.3	5	16.7
Chemotherapy		25	83.3	20	66.7
Previous SRE		9	30.0	13	43.3

Abbreviation: *SD* standard deviation

Inclusion criteria were an age of 18 to 80 years, a Karnofsky performance score [11] ≥ 70, written consent to participate, and already initiated bisphosphonate therapy. Patients were excluded if they presented with concomitant pathologies that could interfere in the evaluation of bone turnover markers, such as bone metabolic disorders, Paget`s disease of bone, hyperparathyroidism, thyroid abnormalities, abnormal intestinal absorption, or hepatic or renal dysfunction. In all cases, anticancer treatment could be changed as clinically indicated throughout the course of the study. The patients were subjected to a staging of their vertebral column within the context of the computed tomography (CT) designed to plan the radiation schedule prior to enrolment into the trial. In this examination metastases were classified as “stable” or “unstable”. This was diagnosed independently by a specialist for radiology as well as by a specialist for orthopedic surgery. The specifications for an unstable vertebral body were tumor occupancy more than 60 % of the vertebral body, and pedicle destruction [12]. Only a metastasis classified by both specialists as “stable” was suggested eligible for inclusion. Patients with significant neurological or psychiatric disorders were excluded. The study was approved by the Heidelberg Ethics Committee (Nr. S-316/2011).

### Radiotherapy

RT was performed in the Radiooncology Department of the Heidelberg University Clinic. After virtual simulation was performed to plan the radiation schedule, RT was carried out over a dorsal photon field of the 6MV energy range. Primary target volume (PTV) covered the specific vertebral body affected as well as the ones immediately above and below. In Arm A, 24 patients (80 %) were treated with 10 × 3 Gy, three patients (10 %) with 14 × 2.5 Gy, and three patients (10 %) with 20 × 2 Gy. In Arm B the dose fractions for 28 patients (93.4 %) were 10 × 3 Gy, for one patient (3.3 %) 14 × 2.5 Gy, and for one patient (3.3 %) 20 × 2 Gy. The median individual dose in all patients was 3 Gy (range 2–3 Gy), the median total dose 30 Gy (range 20–35 Gy). The individual and total doses were decided separately for each individual patient, depending on the histology, the patient’s general state of health, and on the current staging and the corresponding prognosis.

### Study interventions

The interventions commenced on the same day as RT and were performed on each day of RT treatment (Monday through Friday) over a 2-week period, independent of the number of fractions. During the 2-week RT period, the patients in the resistance training arm (Arm A) performed the exercises under the guidance of a physiotherapist. The patients were then instructed to practice the training in their homes three times a week and continued the resistance training themselves until the last investigation after three months, and conducted the documentation in form of a training diary. The resistance training lasted approx. 30 min, the passive physical therapy (Arm B) approx. 15 min. Since the site of the bone metastases differed from patient to patient, three different exercises were enacted to ensure an even isometric resistance training of the paravertebral muscles. Patients in the control arm (Arm B) received passive physical therapy in form of breathing exercises also for a period of 2 weeks. This was conducted so that these patients were not discouraged in comparison to the intervention arm, and for avoidance of a high drop-out rate. A detailed report of the intervention and its application has already been published [13].

### Statistical analysis

Bone marker values were presented in terms of mean and standard deviation (SD). The possible difference between arms at 3 months was calculated using ANCOVA with arm as factor and baseline value as covariate. Therefore as all surviving patients completed all surveys we assumed that 23 patients in Arm A and 24 patients in Arm B were eligible for the analysis. Multivariate binary logistic regression models were fitted to quantify the degree of association between potential predictors of markers at baseline according to local response (yes/no), prevention of SRE (yes/no), and number of metastases (1/more than one). Group had been included as a covariate in the analysis. Arm A with 23 survived patients was the reference. Level of significance was set to 0.05 for all tests. All statistical analyses were done using SAS software Version 9.4 (SAS Institute, Cary, NC, USA).

### Analytic methods

Samples were centrifuged at 3.500 rpm for 10 min after drawing, serum obtained and stored at −80°. First morning void of urine was collected, mixed and an aliquot of 10 ml stored at – 80 °C. Samples shipped on dry ice to the laboratory (Labor Limbach, Heidelberg, Germany) and again stored at −80 °C until analysis.

Total aminoterminal propeptide of type I collagen (PINP; bone formation; interassay coefficient of variation [CV]: 2.7 %), C-terminal cross-linking telopeptide of type I collagen (CTX-I; bone resorption; CV: 3.1 %) and intact parathyroid hormone (iPTH; CV: 1.4 %) were measured by means of an automated electrochemiluminescence immunoassay (ECLIA; Modular Analytics E170, Roche Diagnostics, Penzberg, Germany). Bone-specific alkaline phosphatase (BAP; bone formation; CV: 5,2 %) was measured by means of a spectrophotometric immunoassay (IDS-ISYS Ostase BAP; Immunodiagnostic Systems Ltd [IDS Ltd], Boldon, Tyne & Wear, UK) on the fully automated immunoassay system (IDS-ISYS (Immunodiagnostic Systems Ltd [IDS Ltd], Boldon, Tyne & Wear, UK). Pyridinoline (PYD) and deoxypyridinoline (DPD; bone resorption; CV: 12.5 %) were assayed by high performance liquid chromatography (HPLC) [14, 15]. Analysis required first a sample extraction, hydrolysis and automated column-chromatographic pre-purification. To avoid any effect of different dilutions of urine, Pyr and Dpyr data are expressed against gram of creatinine. Cross-linked N-telopeptides of type I collagen (NTx; bone resorption; CV: 12.8 %) was determined with an commercially available ELISA (Osteomark Ntx; Wampole Laboratories, Princeton NJ, USA).

All laboratory analyses were done in two batches after completion of the sample collection. To further reduce imprecision of measurement, all samples were analyzed utilizing one reagent lot. Tests and instruments were run strictly in accordance with the guidelines given by the manufacturer and were subject to continuous maintenance and service according to the laboratories standard operating procedures for good laboratory practice. Samples were thawed at the day of analysis at ambient temperature, mixed on a head-over-head mixer and centrifuged before measurement.

## Results

**Fig. 1:**
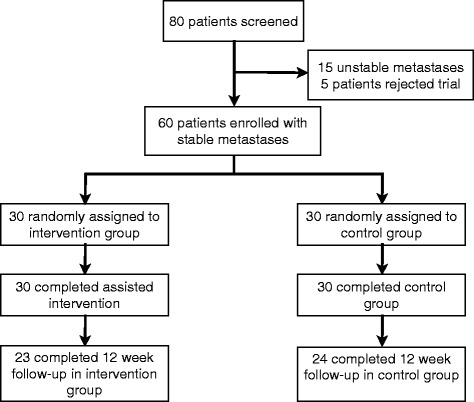
Flow of participants through the trial

From September 2011 to March 2013, consecutive 80 patients with a histologically confirmed cancer of any primary and spinal bone metastases of the thoracic or lumbar segments, or of the sacral region were considered in the Radiooncology Department of the Heidelberg University Clinic. Fifteen patients were excluded due to unstable metastases, and five patients declined to participate in the study. Sixty patients fulfilled the inclusion and were enrolled into the trial (Fig. 1). Arms were balanced at baseline without group differences, particularly number of metastases and tumor size (Table 1). Seven patients (23.3 %) died in arm A within the first 12 weeks following RT, six patients in arm B (20.0 %) died within 3 months. The mean follow-up was 3.3 months for both groups. All surviving patients completed all surveys. Mortality did not differ between groups.

Mean change values of PYD and CTX-I were significantly lower at 3 months after RT (*p* = 0.035 and *p* = 0.043) in arm A. DPD showed a tendency with a lower mean change value, but not significant. No changes were seen between the arms in BAP, NTX, and PINP. Importantly, all markers decreased in both arms, except of PYD and CTX-I in arm B, although significance was not reached for some biomarkers (Table 2).

**Table 2:** Results of bone turnover markers

		Arm A				Arm B				Difference between arms	
		baseline, *n*=23		3 months, *n*=23		baseline, *n*=24		3 months, *n*=24		LS means	
Parameter	Reference value	mean	SD	mean	SD	mean	SD	mean	SD	Difference (95 % CI)	*p*-value*
PYD	160–280 μg/g Cr	375.63	206.20	299.89	173.88	402.00	199.01	405.50	187.32	−90.92 (−175.19; −6.65)	0.035
DPD	26–65 μg/g Cr	67.30	39.64	51.20	35.81	86.10	78.85	72.88	37.11	−18.20 (−38.84; 2.45)	0.082
CTX-I	<0.5484 ng/ml	0.29	0.27	0.20	0.23	0.39	0.37	0.42	0.45	−0.10 (−0.20; −0.003))	0.043
BAP	6–15 μg/l	56.89	92.31	31.46	63.31	44.17	47.32	36.52	37.58	−12.10 (−31.51; 7.31)	0.215
NTX	5.4–24.2 nmol/l	19.09	7.21	17.42	7.98	21.42	9.36	20.38	9.81	−0.87 (−3.84; 2.10)	0.556
PINP	15–59 μg/l	67.54	40.69	49.50	41.43	87.96	51.98	66.77	39.18	−6.94 (−26.81; 12.93)	0.485

*Results of ANCOVA

This table shows the results of bone turnover markers of both groups at baseline and 3 months after RT. Differences are presented within and between groups

Abbreviation: *LS means* least square means

In arm A, the local response was significantly higher [73.9 % (*n* = 17) vs. 25 % (*n* = 6)] (*p* = 0.003). At first, PINP could be identified as a predictor of local response (*p* = 0.043). BAP (*p* = 0.034) and PINP (*p* = 0.044) could be identified as a predictor for avoidance of SRE. No marker could be detected for solitary metastasis (Table 3).

**Table 3:** Results of multivariate binary logistic regression analysis of bone markers

Local response (no local response)	OR	95 % CI (OR)	*p*-value
Arm (A – reference)	0.033	0.004 – 0.311	0.003
PYD	1.000	0.992 – 1.008	0.986
DPD	1.023	0.974 – 1.074	0.371
CTX-I	10.550	0.003 – >100	0.569
BAP	1.011	0.996 – 1.025	0.142
NTX	0.870	0.688 – 1.101	0.245
PINP	0.968	0.938 – 0.999	0.043
Prevention of SRE (SRE = 0)	OR	95 % CI (OR)	*p*-value
Arm (A – reference)	3.302	0.877 – 12.431	0.077
PYD	0.998	0.994 – 1.002	0.373
DPD	1.006	0.982 – 1.031	0.633
CTX-I	0.323	0.001 – 81.336	0.689
BAP	0.974	0.950 – 0.998	0.034
NTX	0.988	0.861 – 1.134	0.865
PINP	1.025	1.001 – 1.049	0.044
Solitary metastasis (*n* = 1)	OR	95 % CI (OR)	*p*-value
Arm (A – reference)	1.692	0.477 – 6.007	0.416
PYD	1.001	0.997 – 1.005	0.512
DPD	1.004	0.980 – 1.029	0.761
CTX-I	0.359	0.001 – >100	0.723
BAP	0.997	0.985 – 1.009	0.592
NTX	1.083	0.939 – 1.249	0.275
PINP	0.998	0.981 – 1.016	0.829

Abbreviation: *SRE* skeletal-related events

## Discussion

Bone metastases are a very frequent secondary diagnosis associated with an advanced tumor disease, with the vertebral column being the most frequent localization [2, 10]. The usefulness of BTM as tool for the diagnosis of bone metastases in many types of cancers has been investigated previously [3]. Many studies have analyzed their relationship with clinical outcomes [7, 16] as well as their clinical importance [17, 18]. However, there is still no concensus among these studies as to which BTM is the ideal marker.

In the current work, all BTM in arm A decreased after 3 months, especially the mean change values of PYD and CTX-I were significantly lower at 3 months after RT due to resistance training. BTM in arm B showed also decreased values after 3 months except of PYD and CTX-I, but not so distinctive as in arm A. Importantly, local response as re-calcification in metastasis was improved in arm A due to resistance training. In our recent work, we could show that bone density was significantly increased in metastasis 3 months after start of resistance training concomitant to RT [19]. This factor may influence the BTM and was demonstrated in our results. Therefore, PINP could be detected as a strong marker for bone formation after combined treatment with resistance training concomitant to RT.

Determination of CTX-I is recommended for monitoring the efficacy of antiresorptive therapy. Patients with renal cell carcinoma who died or progressed had higher baseline B-CTX levels and those who experienced SRE during follow-up showed high BAP levels [20]. However, in our results we could not found any useful data in normal/abnormal values of BTM. A multicenter study found that increased values of BAP and NTX were associated with increased risk of SRE, and disease progression in patients with breast cancer, prostate cancer, and other solid tumors, treated with bisphosphonates [21]. These markers of bone turnover have been used clinically to predict the risk of SREs and disease outcomes. According our results, we could identify BAP and PINP for avoidance of SRE. In addition, biochemical markers of bone turnover may prove useful in the monitoring of patients on bisphosphonate treatment for bone metastases. One review [22] of 121 patients with skeletal metastases from variety of solid tumors revealed that mean values of NTX were consistently elevated in patients experiencing SREs compared with those who did not. An additional study [23] showed in 441 patients that patients with high NTX or BAP levels had a greater incidence of SREs compared with patients with low levels of NTX or BAP. Patients with increased NTX or BAP at baseline had an increased risk for SRE, shorter time to first event, disease progression and death. As NTX levels have been shown to correlate with response to bisphosphonate treatment. In our data, we could not show any correlation of NTX to local response, SRE, and number of metastases, but we were able to confirm the relation to BAP with prevention of SRE. Zoledronic acid normalizes or maintains normal NTX levels in most patients with bone metastases. All of our participants were treated with bisphosphonates. Our data presented no differences between arms of NTX.

However, a PINP could be identified as a predictor for local response and avoidance of SRE. In this novel study, our results showed that resistance training concomitant to RT can even enhance an effect on BTM.

Since all patients were in advanced stages of their cancer, 40 % of the patients in either group were lost to follow-up due to progressive disease and subsequently death. Further limitations of the study were the relatively small sample size, the variety of primary tumors and patient conditions, and the exclusion of patients presenting with cervical spine metastases.

Among the strengths of our novel and original study were the randomized design and the very first presentation of bone turnover markers in combined treatment of resistance training concomitant to RT in patients with spinal bone metastases.

## Conclusion

In this group of patients with spinal bone metastases, we were able to show that patients with guided resistance training of the paravertebral muscles can influence BTM. PYD and CTX-I decreased significantly in arm A. PINP can be considered as a complementary tool for prediction of local response, and PINP as well as BAP for avoidance of SRE. More studies in larger groups of patients are necessary for further confirmatory research.

## Ethics approval

The study was approved by the Heidelberg Ethics Committee (Nr. S-316/2011).

## Consent for publication

Not applicable.

## Availability of data and materials

The dataset supporting the conclusions of this article is included within the article

## Abbreviations

ANCOVA: analysis of covariance
BAP: bone alkaline phosphatase
BTM: bone turnover markers
CT: computed tomography
CTX-I: beta-isomer of carboxy-terminal telopeptide of type I collagen
CV: coefficient of variation
DPD: desoxy-pyridinoline
ECLIA: automated electrochemiluminescence immunoassay
Gy: Gray
HPLC: high performance liquid chromatography
iPTH: intact parathyroid hormone
NTX: cross-linked N-telopeptide of type I collagen
PINP: total amino-terminal propeptide of type I collagen
PTV: Primary target volume
PYD: pyridinoline
RT: radiation therapy
SD: standard deviation
SRE: skeletal related events
